# Clinicopathological Characteristics of Gynecological Cancer Associated with Hypoxia-Inducible Factor 1α Expression: A Meta-Analysis Including 6,612 Subjects

**DOI:** 10.1371/journal.pone.0127229

**Published:** 2015-05-19

**Authors:** Yue Jin, Haolu Wang, Xiaowei Ma, Xiaowen Liang, Xin Liu, Yu Wang

**Affiliations:** 1 Department of Gynecology, Ren Ji Hospital, School of Medicine, Shanghai Jiao Tong University Shanghai, China; 2 Department of Biliary-Pancreatic Surgery, Ren Ji Hospital, School of Medicine, Shanghai Jiao Tong University, Shanghai, China; 3 Therapeutics Research Centre, Princess Alexandra Hospital, School of Medicine, The University of Queensland, Brisbane, Australia; 4 Department of Clinical Laboratory, Ren Ji Hospital, School of Medicine, Shanghai Jiao Tong University, Shanghai, China; University of North Carolina School of Medicine, UNITED STATES

## Abstract

**Background:**

Gynecological cancer is characterized by tumor hypoxia. However, the role of hypoxia-inducible factor 1α (HIF-1α) in gynecological cancer remains unclear.

**Method:**

Electronic databases including Cochrane Library, PUBMED, Web of Knowledge and clinical trial registries were searched from inception through October 2014 for published, case-control studies assessing the association between HIF-1α and the clinicopathological characteristics of gynecological cancer. We pooled results from 59 studies using fixed or random-effects models and present results as odds ratios (ORs) following the PRISMA guidelines.

**Results:**

Our meta-analysis, which included 6,612 women, demonstrated that the expression of HIF-1α was associated with the clinicopathological characteristics of gynecological cancer. The expression of HIF-1α in cancer or borderline tissue was significantly higher than that in normal tissue (cancer vs. normal: odds ratio (OR) =9.59, 95% confidence interval (CI): 5.97, 15.39, p<0.00001; borderline vs. normal: OR=4.13, 95% (CI): 2.43, 7.02, *p*<0.00001; cancer vs. borderline: OR=2.70, 95% (CI): 1.69, 4.31, *p*<0.0001). The expression of HIF-1α in III‒IV stage or lymph node metastasis was significantly higher than that in I‒II stage or that without lymph node metastasis, respectively (OR=2.66, 95% (CI): 1.87,3.79, *p*<0.00001; OR= 3.98, 95% (CI): 2.10,12.89, *p*<0.0001). HIF-1α was associated with histological grade of cancer (Grade 3 vs. Grade 1: OR=3.77, 95% (CI): 2.76,5.16, *p*<0.00001; Grade 3 vs. Grade 2: OR=1.62, 95% (CI): 1.20,2.19, *p*=0.002; Grade 2 vs. Grade 1: OR=2.34, 95% (CI): 1.82,3.00, *p*<0.00001),5-years disease free survival (DFS) rates (OR=2.93, 95% (CI):1.43,6.01, *p*=0.001) and 5-years overall survival (OS) rates (OR=5.53, 95% (CI): 2.48,12.31, *p*<0.0001).

**Conclusion:**

HIF-1α is associated with the malignant degree, FIGO stage, histological grade, lymph node metastasis, 5-years survival rate and recurrence rate of gynecological cancer. It may play an important role in clinical treatment and prognostic evaluation.

## Introduction

Solid tumors outgrow their own vasculature beyond the size of several cubic millimeters, resulting in hypoxia. HIF-1 regulates cellular oxygen homeostasis, and plays a key role in hypoxic conditions that occur during tumor angiogenesis, invasion and metastasis [1, 2]. HIF-1 is a heterodimeric transcription factor that consists of α and β subunits. The β subunit is constitutively expressed, while the expression of HIF-1α is regulated by the oxygen level [3]. Under normoxic conditions, HIF-1α would be degraded due to targeted ubiquitination and degradation by the proteasome. This process is mediated by direct binding of von Hippel—Lindau tumor suppressor protein (pVHL), a component of the E3 ubiquitin—protein ligase complex, with the minimal N-terminal transactivation domain (N-TAD) located within the oxygen-dependent degradation domain of HIF-1α. On the contrary, in hypoxic conditions, the degradation of HIF-1α is suppressed and the expression of HIF-1α would increase. Over-expression of HIF-1α has been reported in many types of malignancies, including lung, prostate, breast, colon and rectum carcinoma, and in both regional and distant metastases, implying that HIF-1α may play a vital role in tumor progression [4–6].

Gynecological malignancies, including cancers of endometrium, cervix, ovary, vulva and vagina, account for 11.7% of all new cancers in women. The American Cancer Society estimates that 94,990 women will have been diagnosed with, and 28,790 women will have died of, cancer of the female genital tract in 2014 in the USA [7]. Thus, it is important to understand the mechanisms of carcinogenesis and progression in gynecological cancer. HIF-1α is a key cellular survival protein during hypoxia, and is associated with tumor progression and metastasis in various solid tumors. In gynecological malignancies, Birner *et al*. [8] suggested that HIF-1α was a facilitator of premalignant progression. Acs *et al*. [9] and Birner *et al*. [10] found a consistent correlation between tumor stage and HIF-1α expression. Moreover, Seeber *et al*. [11], Bachtiary *et al*. [12] and Shimogai *et al*. [13] proposed HIF-1α as a predictor of poor prognosis and response to therapy. However, results of studies on HIF-1α in gynecological cancer are not always consistent. We carried out the first meta-analysis to assess the potential association between HIF-1α and the clinicopathological parameters of gynecological cancer. Cancers of the vulva and vagina are relatively rare. No study on HIF-1α and the clinicopathological characteristics of these malignancies has been published. Cancers of endometrium, cervix and ovary were included as subgroups in the final analysis.

## Materials and Methods

### Search strategy

We conducted the literature searches and meta-analysis following the Preferred Reporting Items for Systematic Reviews and Meta-analyses (PRISMA) guidelines (S1 PRISMA Checklist). The electronic databases including Cochrane Library, PUBMED, Web of Knowledge and clinical trial registries, were used for systematic literature searches. Eligibility was restricted to studies published from inception to October 2014 with abstract or full text available. No language restrictions were made. We employed “hypoxia- inducible factor”, “HIF-1α”, or “HIF-1”, concatenated with “gynecological”, “endometrial”, “cervical”, “ovarian”, “vulva”, “vagina” and “tumor”, “cancer”, “carcinoma”, or “malignancy” as search terms. A comprehensive search of reference lists of all review articles and original studies retrieved by this method was performed to identify additional reports.

### Criteria for inclusion and exclusion

The inclusion criteria for primary studies were as follows: (1) primary gynecological cancer should be pathologically proven; and (2) HIF-1α expression should be detected with immunohistochemistry (IHC); and (3) the association between clinicopathologic variables and HIF-1α expression should be described; or (4) provides information on survival data; and (5) laboratory methodology of IHC: (5.1) the staining of protein should be described (nuclear, cytoplasm); and (5.2) tissue sample conservation (fixation in formalin, alcohol or paraffin); and (5.3) description of the revelation test procedure of the biological factors with the first antibody type, clone identification, second antibody type, reaction characteristics, coloration method and epitope unmasking method; and (5.4) description of the negative and positive control; and (5.5) definition of the level of positivity of the test; or (5.6) the pathologist evaluating the IHC outcome was double-blind (or random) to patient clinicopathologic data and outcome. When studies were retrospective, the pathologist blinding was simple-blind.

Exclusion criteria for primary studies were as follows: (1) review, abstract, case report, animal or cell studies; or (2) not possible to extract the exact data (the association between clinicopathologic variables and HIF-1α expression); or (3) patients received chemotherapy, radiotherapy, targeted therapy before operation; and (4) laboratory methodology of IHC: (4.1) the study design was not defined; or (4.2) was unclear and no detailed description of standard laboratory methodology about IHC; or (4.3) the pathologist blinding was unblinded.

### Review procedure and data extraction

Titles and abstracts were studied to assess inclusion criteria and examined independently for eligibility by two reviewers (Y. Jin and H. Wang). Disagreements were resolved by consulting a third reviewer (Y. Wang). The study characteristics were recorded as follows: (1) the first author, the nationality of included patients, article publication year; (2) the number of patients, cancer cases, borderline cases and controls for positive HIF-1α expression (HIF-1α expression score ≥ +), which was measured by semi-quantitatively assessing the percentage of tumor cells expressing HIF-1α, intensity of cell staining and extent of staining; (3) the number of test cases (FIGO III–IV stage, lymph nodes metastasis) and control cases (FIGO I–II, no lymph nodes metastasis) for positive HIF-1α expression; (4) the number of test cases (Grade 3 or Grade 2) and control cases (Grade 1); (5) the hazard ratio of 5-year disease free survival (DFS) and OS.

### Quality assessments

Newcastle-Ottawa Scale (NOS) was used to assess the methodological quality of the included case-control studies. A study can be awarded 1 point for each numbered item in nine of NOS. Studies with scores of 0–4 are considered as low-quality, while 5–9 as high-quality.

### Statistical analysis

We estimated the odds ratio (OR) for clinicopathologic variables (FIGO III–IV vs. FIGO I–II; lymph nodes metastasis vs. no lymph nodes metastasis; Grade 3 or Grade 2 vs. Grade 1), 5-year DFS and 5-year overall survival (OS). Statistical heterogeneity assumption among studies was checked using the X^2^-based Q-test. When *I* ^2^ was less than 50%, pooled odds ratios, relative risk and 95% confidence intervals (CIs) were calculated using Mantel-Haenszel method with fixed effect models. Whereas significant heterogeneity among the studies was detected (*I* ^2^>50%), a random-effect model was adopted. If necessary, a sensitive analysis was also performed to evaluate the influence of individual studies on the final effect. All p-values were two-sided. A *p*-value <0.05 was considered significant. All the statistical analyses were performed using RevMan 5.0 software (The Cochrane Collaboration, Oxford, United Kingdom).

## Results

### Description and quality assessments of included studies

**Fig 1:**
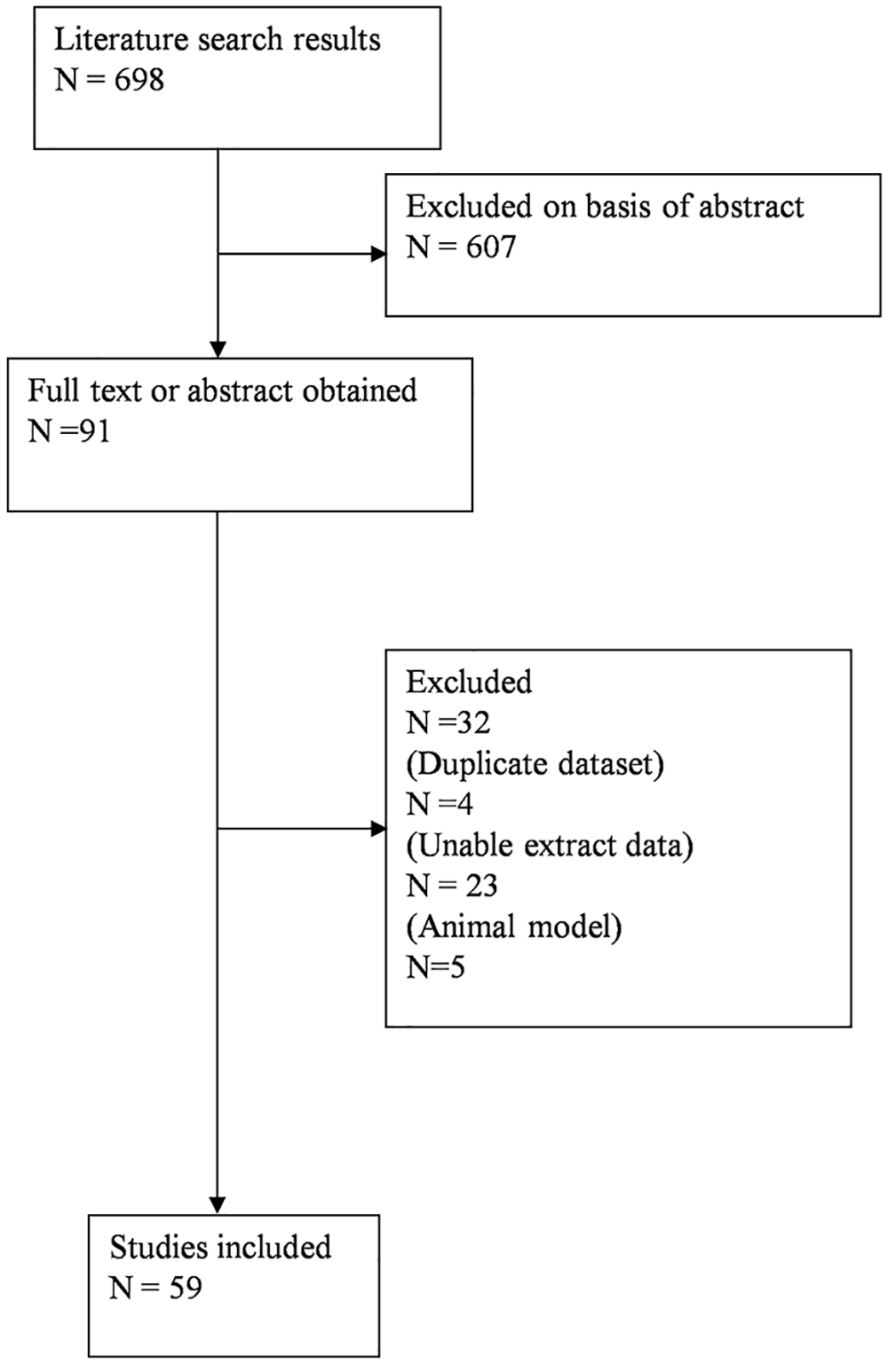
Flowchart of study selection. Sixty independent studies were included in the final review.

The bibliographical search yielded a total of 698 studies and full text or abstract was obtained for 91 studies. Thirty-two of these studies did not meet the inclusion criteria: four studies referred to a duplicate dataset, twenty-three studies did not present exact data to extract, and five was animal studies. Finally, fifty-nine independent studies [2, 8–65] were included in the final review. The processes of study selection were summarized in the flow diagram (Fig 1). The main characteristics of the eligible studies were shown in Table 1, and the quality assessments of the included studies were summarized in S1 Table.

**Table 1:** Characteristics of studies included in this meta-analysis.

Author	Number of patients	Year (country)	HIF-1α positive(negative)	Pathological type	Histological type	FIGO stage	Histological grade	Lymph node metastasis	5-years overall survival rate	5-years disease free survival rate
**Ovarian cancer**				(cancer/borderline/ benign)	(serous/clear cell/others)	(I–II/III–IV)	(G1/G2/G3)	(yes/no)	(<5/≥5)	(<5/≥5)
Daponte^14^	120	2008(Greece)	61(59)	78/22/20	-	-	-	-	-	-
Shimogai^13^	66	2008 (Japan)	11(55)	66/-/-	48/5/13	22/44	-	25/41	24/42	11/55
Yu^15^	117	2012 (China)	59(58)	87/-/30	75/12*	45/44	-	42/45	53/34	-
Birner^10^	172	2001(Austria)	116(56)	102/50/20	64/8/30	-	-	-	-	-
Osada^16^	107	2007 (Japan)	82(25)	72/17/18	-	48/24	32/30/10	-	-	-
Shen^17^	63	2013 (China)	55(8)	63/-/-	-	44/19	19/17/16	-	-	-
Su^18^	81	2011 (China)	40(41)	35/22/24	-	13/22	4/17/14	-	-	-
Yu^19^	30	2009 (China)	26(4)	30/-/-	18/2/10	12/18	10/10/8	-	-	-
Liu^20^	171	2012 (China)	80(91)	96/-/45	45/8/43	30/66	24/40/32	-	-	-
Chen^21^	62	2011 (China)	29(33)	62/-/-	40/22*	26/36	25/37^△^	36/26	44/18	-
Fu^22^	119	2008 (China)	70(49)	101/-/-	51/9/41	53/48	-	-	-	-
Guo^23^	108	2010 (China)	39(66)	58/-/30	-	20/38	18/28/12	27/31	-	-
Naka^26^	52	2007 (Japan)	36(16)	52/-/-	29/9/14	-/52	19/14/10	-	-	-
Ji^25^	116	2013 (China)	70(46)	41/20/27	-	20/21	-	27/14	-	-
Nakayama^26^	60	2002 (Japan)	30(30)	60/-/-	29/17/14^#^	23/37	17/16/22	-	-	-
Iida^27^	102	2008 (Japan)	91(11)	39/32/31	-	-	-	-	-	-
Chen^28^	164	2012 (China)	62(102)	124/-/-	80/44^▲^	53/71	49/75^△^	50/74	-	-
Li^29^	141	2011(China)	66(75)	60/21/30	40/20*	19/41	23/37	36/24	-	-
Wong^30^	53	2003(USA)	22(31)	37/-/16	29/2/6	-/37	-	-	-	-
Luo^31^	308	2005(China)	208(100)	238/19/38	148/20/70	77/161	53/101/84	-	-	-
Wang^32^	145	2008(China)	86(79)	112/9/18	58/33/31^#^	46/76	24/48/38	-	-	-
Tong^34^	31	2008(China)	26(5)	31/-/-	31/-/-	-	-	21/10	-	21/10
Li^33^	73	2009(China)	35(38)	37/19/-	-	13/24	12/25^$^	27/10	-	-
Miyazawa^35^	36	2009(Japan)	21(2)	23/2/11	5/7/11	-	-	-	-	-
Yasuda^36^	74	2008(Japan)	69(5)	74/-/-	21/18/35	-	-	-	-	-
**Cervical cancer**				(cancer/CIN/normal)	(squamous/ others)	(I–II/III–IV)	(G1/G2/G3)	(yes/no)	(<5/≥5)	(<5/≥5)
Cheng^37^	158	2013(China)	63(35)	98/32/28	98/-	57/41^@^	42/35/21	39/59	-	-
Kim^38^	745	2013(Korea)	60(91)	179/209/357	144/35	174/5	-	-	17/134	31/120
Huang^39^	74	2014(China)	39(35)	74/-/-	58/16	35/39^$^	38/36^△^	17/57	-	-
Dellas^40^	44	2008(Germany)	32(12)	44/-/-	-	9/35	-	-	19/25	-
Birner^8^	106	2000(Austria)	20(71)	91/10/5	-	91/-	-	-	17/74	28/63
Bachtiary^12^	67	2003(Austria)	32(35)	67/-/-	59/8	40/27	7/34/17	21/46	-	-
Li^41^	120	2010(China)	90(30)	40/40/40	40/-	40/-	10/21/9	10/30	-	-
Guo^42^	189	2008(China)	93(96)	79/90/20	79/-	54/25	17/36/26	-	-	-
Liu^43^	93	2008(China)	26(19)	45/28/20	45/-	45/-	29/16^△^	-	-	-
Zhang^44^	54	2009(China)	28(26)	34/10/10	23/11	34/-	13/21^$^	19/15	-	-
Acs^45^	170	2003(USA)	143(27)	15/70/85	15/-	15/-	-	-	-	-
Hutchison^46^	99	2004(United Kingdom)	68(31)	99/-/-	-	57/42	17/57/14	-	-	-
No^47^	116	2009(Korea)	40(76)	36/39/41		-	-	11/25	-	-
Ishikawa^48^	38	2004(Japan)	20(18)	38/-/-	38/-	-/38	-	-	-	17/21
Haugland^49^	101	2002(Canada)	23(22)	45/-/-	33/12	30/15	-	11/34	-	-
Burri^50^	91	2003(Switzerland)	46(32)	78/-/-	63/15	9/43/26^&^	-	30/47	-	-
Markowska^51^	106	2007(Poland)	81(25)	106/-/-	106/-	-	29/46/31	-	-	-
**Endometrial cancer**				(cancer/borderline/ normal)	(type 1/ type 2)	(I–II/III–IV)	(G1/G2/G3)	(yes/no)	(<5/≥5)	(<5/≥5)
Ozbudak^52^	100	2008(Turkey)	45(55)	100/-/-	100/-	69/31	60/25/15	-	-	-
Feng^53^	187	2013(China)	100(87)	124/28/35	124/-	101/23	57/41/26	31/93	-	-
Espinosa^54^	64	2010(Italy)	17(32)	64/-/-	64/-	24/25	14/22/28	-	-	-
Seeber^69^	108	2010(Netherlands)	54(39)	93/-/-	75/18	75/18	28/47/18	-	-	18/72
Pijnenborg^55^	65	2007(Netherlands)	14(51)	65/-/-	65/-	60/5	20/29/16	-	-	40/25
Acs^9^	166	2004(USA)	79(28)	107/-/59	74/33	65/42	36/20/51	-	-	-
Pansare^56^	149	2007(USA)	54(90)	149/-/-	80/41	114/30	42/66^$^	-	-	-
Horrée^57^	79	2007(Netherlands)	48(31)	39/23/17	39/-	23/16	6/21/12	-	-	-
Koda^58^	85	2007(Poland)	55(30)	60/-/25	-	29/31	8/44/8	-	-	-
Aybatli^59^	94	2011(Turkey)	28(66)	94/-/-	76/18	64/30	36/30/28	34/60	-	9/85
Yeramian^60^	93	2011(Spain and USA)	26(55)	93/-/-	93/-	-	26/35/21	-	-	9/72
Li^61^	54	2008(China)	20(34)	42/-/12	36/6	21/21	8/34^$^	32/10	-	-
Zhai^62^	62	2007(China)	25(37)	42/-/20	42/-	28/14	25/17^△^	16/26	-	-
Pan^63^	93	2011(China)	51(42)	52/23/18	52/-	32/20	17/17/18	11/41	-	-
Song^64^	40	2009(China)	26(14)	30/10/-	20/10	27/3	-	-	-	-
Sivridis^2^	106	2002(Greece)	40(41)	81/-/25	81/-	81/-	50/31^$^	-	10/71	-
Wang^65^	125	2010(China)	65(33)	105/-/20	105/-	92/13	53/40/12	-	12/86	-

*: serous/mucinous;

^#^: serous/mucinous/others;

^▲^: serous/others;

^△^: G_1_-G_2_/G_3_;

^$^: G_1_/G_2_-G_3_;

^@^: Ia_1_-IIa/IIb-IIIb; $: Ia_2_-Ib_1_/Ib_2_-IIb; &: Ib-IIa/IIb-IIIa/IIIb-IVa

### HIF-1α expression and pathological variables

All 59 studies including 6612 patients explored the association between HIF-1α expression and clinicopathological variables of gynecological cancer. We performed pooled analyses with available data on the association between HIF-1α expression and pathological type, FIGO stage, histological type, and lymph node metastasis. Table 2 summarized the evaluations of association between HIF-1α expression and clinicopathological variables of gynecological cancer.

**Table 2:** Quantitative analyses of HIF-1α expression and clinicopathological variables of gynecological cancer.

Variables	Number of patients	Test of association			Test of heterogeneity			Meta-analysis model
		OR (95% CI)	Z test	*p* value	Q	*p* value	*I* ^2^ (%)	
**Pathological type**								
Cancer vs Borderline								
Endometrial cancer	212	4.45[2.57,7.71]	5.33	<0.00001	2.36	0.50	0	Fixed
Cervical cancer	328	2.36[1.04,5.38]	2.05	0.04	18.09	0.003	72	Random
Ovarian cancer	1045	2.31[1.04,5.09]	2.07	0.04	63.13	<0.0001	76	Random
Total	1900	2.70[1.69,4.31]	4.15	<0.0001	63.13	<0.0001	70	Random
Cancer vs Normal								
Endometrial cancer	486	11.03[6.55,18.58]	9.02	<0.00001	8.73	0.12	43	Fixed
Cervical cancer	484	8.17[2.80,23.85]	3.85	0.0001	21.59	0.003	68	Random
Ovarian cancer	1401	9.73[4.90,19.32]	6.51	<0.00001	44.90	<0.0001	73	Random
Total	2371	9.59[5.97,15.39]	9.36	<0.00001	76.80	<0.0001	66	Random
Borderline vs Normal								
Endometrial cancer	144	3.48[0.75,16.15]	1.59	0.11	5.43	0.07	63	Random
Cervical cancer	520	2.40[1.52,3.78]	3.78	0.0002	7.59	0.27	21	Fixed
Ovarian cancer	438	6.29[2.69,14.73]	4.24	<0.0001	21.57	0.0006	63	Random
Total	1087	4.13[2.43,7.02]	5.24	<0.00001	41.82	0.0007	59	Random
**FIGO stage**								
Endometrial cancer	830	2.76[1.25,6.09]	2.50	0.01	38.44	<0.0001	74	Random
Cervical cancer	290	1.76[1.03,2.99]	2.08	0.04	3.74	0.29	20	Fixed
Ovarian cancer	1354	3.01[1.92,4.74]	4.78	<0.00001	39.80	0.0008	60	Random
Total	2474	2.66[1.87,3.79]	5.42	<0.00001	83.78	<0.0001	63	Random
**Histological type**								
G3 vs G1								
Endometrial cancer	301	2.65[1.53,4.59]	3.49	0.0005	7.35	0.20	32	Fixed
Cervical cancer	240	4.29[2.26,8.14]	4.46	<0.00001	10.76	0.06	54	Fixed
Ovarian cancer	466	4.52[2.79,7.31]	6.13	<0.00001	16.50	0.06	45	Fixed
Total	1007	3.77[2.76,5.16]	8.32	<0.00001	36.18	0.02	42	Fixed
G3 vs G2								
Endometrial cancer	299	1.15[0.65,2.01]	0.48	0.63	3.33	0.65	0	Fixed
Cervical cancer	347	1.62[0.91,2.90]	1.65	0.10	5.59	0.35	11	Fixed
Ovarian cancer	567	2.02[1.27,3.19]	2.99	0.003	13.91	0.13	35	Fixed
Total	1213	1.62[1.20,2.19]	3.14	0.002	24.17	0.29	13	Fixed
G2 vs G1								
Endometrial cancer	410	2.19[1.43,3.37]	3.58	0.0003	8.23	0.14	39	Fixed
Cervical cancer	351	2.40[1.46,3.93]	3.46	0.0005	3.68	0.60	0	Fixed
Ovarian cancer	541	2.43[1.65,3.59]	4.48	<0.00001	10.41	0.32	14	Fixed
Total	1302	2.34[1.82,3.00]	6.68	<0.00001	22.43	0.38	6	Fixed
**Lymph node metastasis**								
Endometrial cancer	454	4.02[1.32,12.26]	2.44	0.01	10.75	0.03	63	Random
Cervical cancer	471	2.94[1.19,7329]	2.33	0.02	24.73	0.0008	72	Random
Ovarian cancer	566	5.20[2.10,12.89]	3.56	0.0004	33.87	<0.0001	76	Random
Total	1391	3.98[2.10,12.89]	5.00	<0.0001	3.98	<0.0001	71	Random
**5-years desease free survival rate**								
Endometrial cancer	330	1.56[0.36,6.83]	0.60	0.55	11.80	0.008	75	Random
Cervical cancer	280	5.28[2.90,9.63]	5.43	<0.00001	1.91	0.38	0	Fixed
Ovarian cancer	97	2.42[0.80,7.36]	1.56	0.12	0.36	0.55	0	Fixed
Total	707	2.93[1.43,6.01]	2.93	0.003	20.71	0.008	61	Random
**5-years overall survival rate**								
Endometrial cancer	179	3.67[0.52,25.63]	1.31	0.19	2.43	0.12	59	Random
Cervical cancer	286	3.28[1.63,6.60]	3.34	0.008	3.07	0.22	35	Fixed
Ovarian cancer	215	11.46[3.43,38.29]	3.96	<0.0001	4.54	0.10	56	Random
Total	680	5.53[2.48,12.31]	4.19	<0.0001	17.46	0.01	60	Random

**Fig 2:**
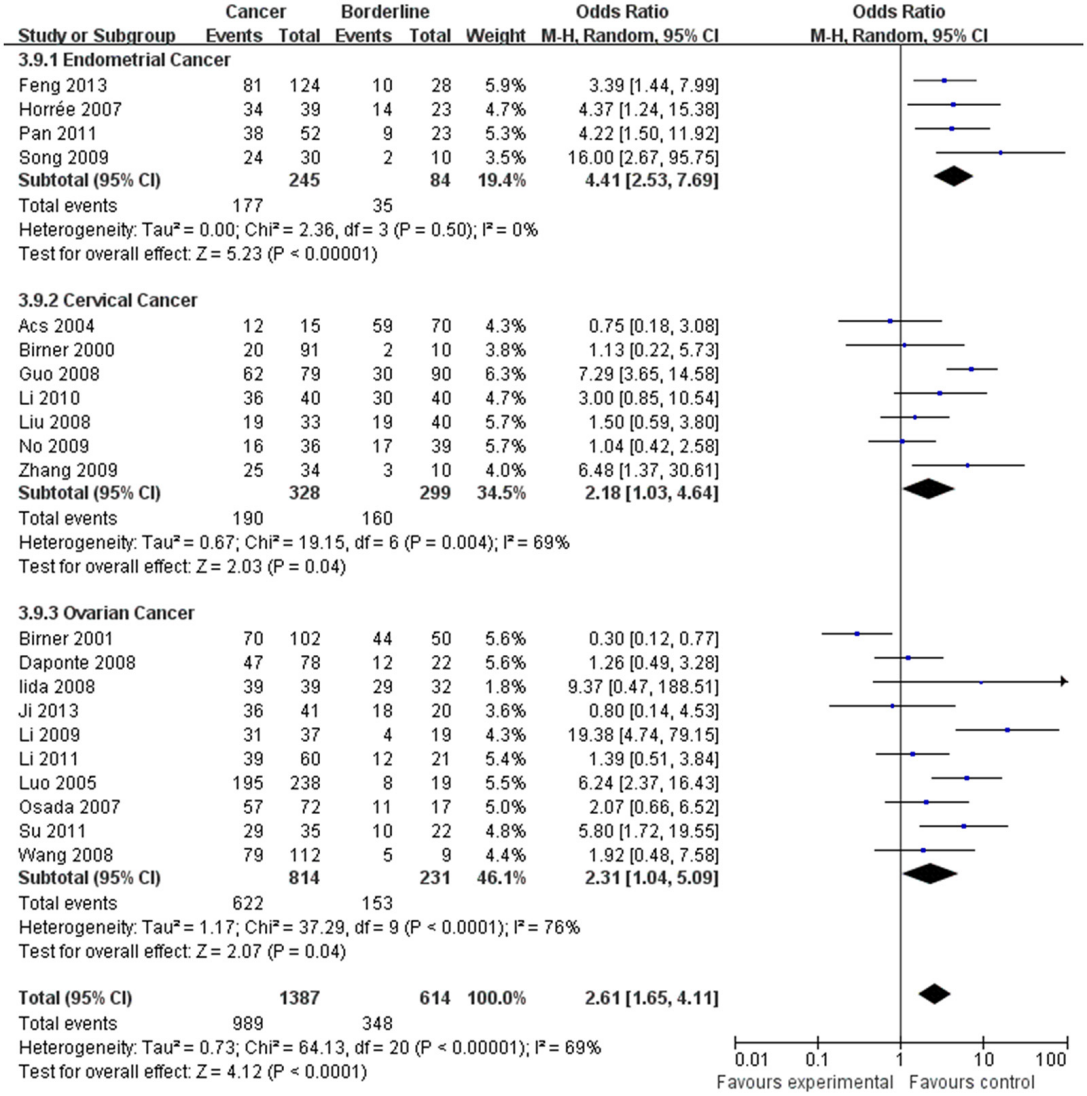
Forest plot of the expression of HIF-1α in cancer versus that in borderline tissue. (*I* ^2^ = 69%).

**Fig 3:**
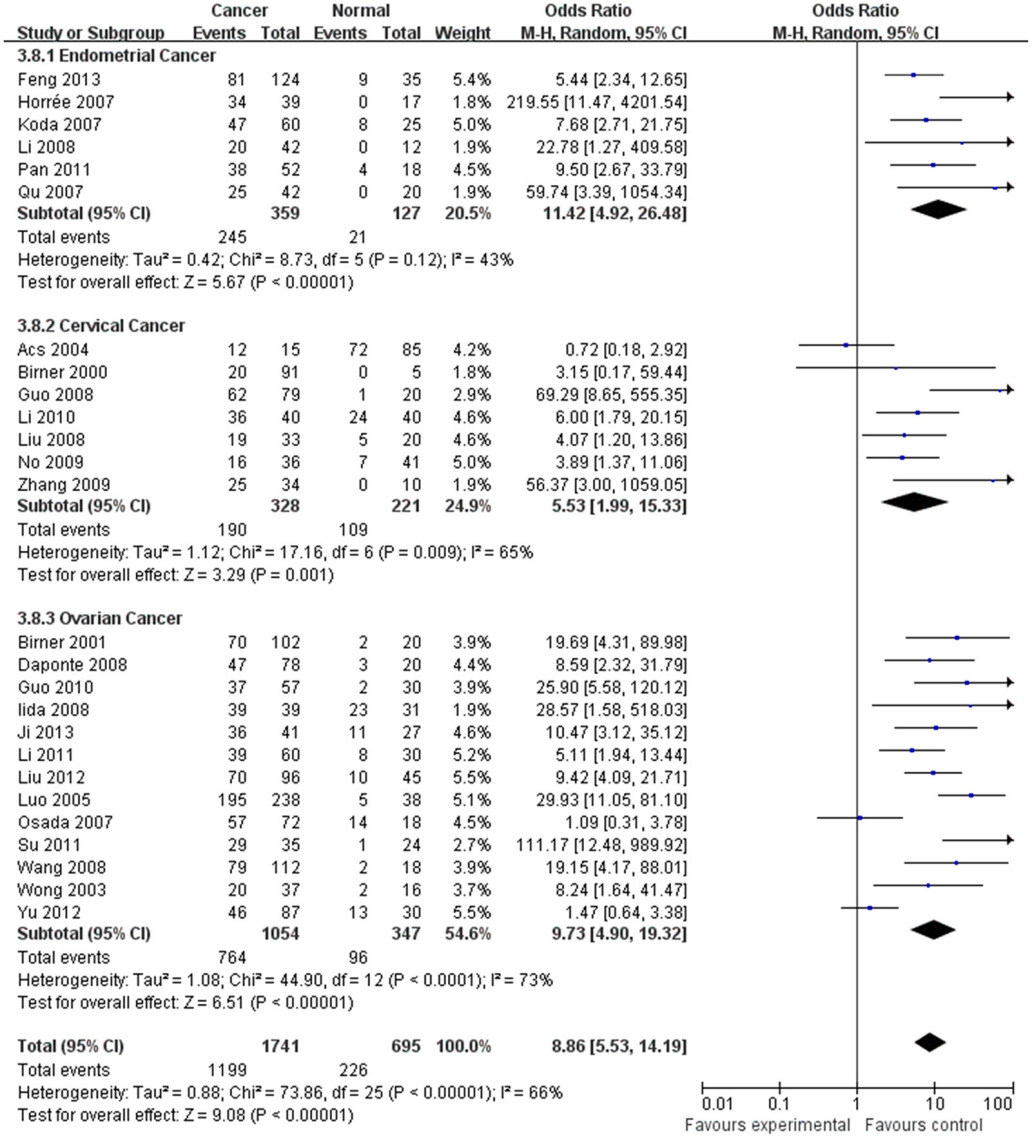
Forest plot of the expression of HIF-1α in cancer versus that in nomal tissue. (*I* ^2^ = 66%).

**Fig 4:**
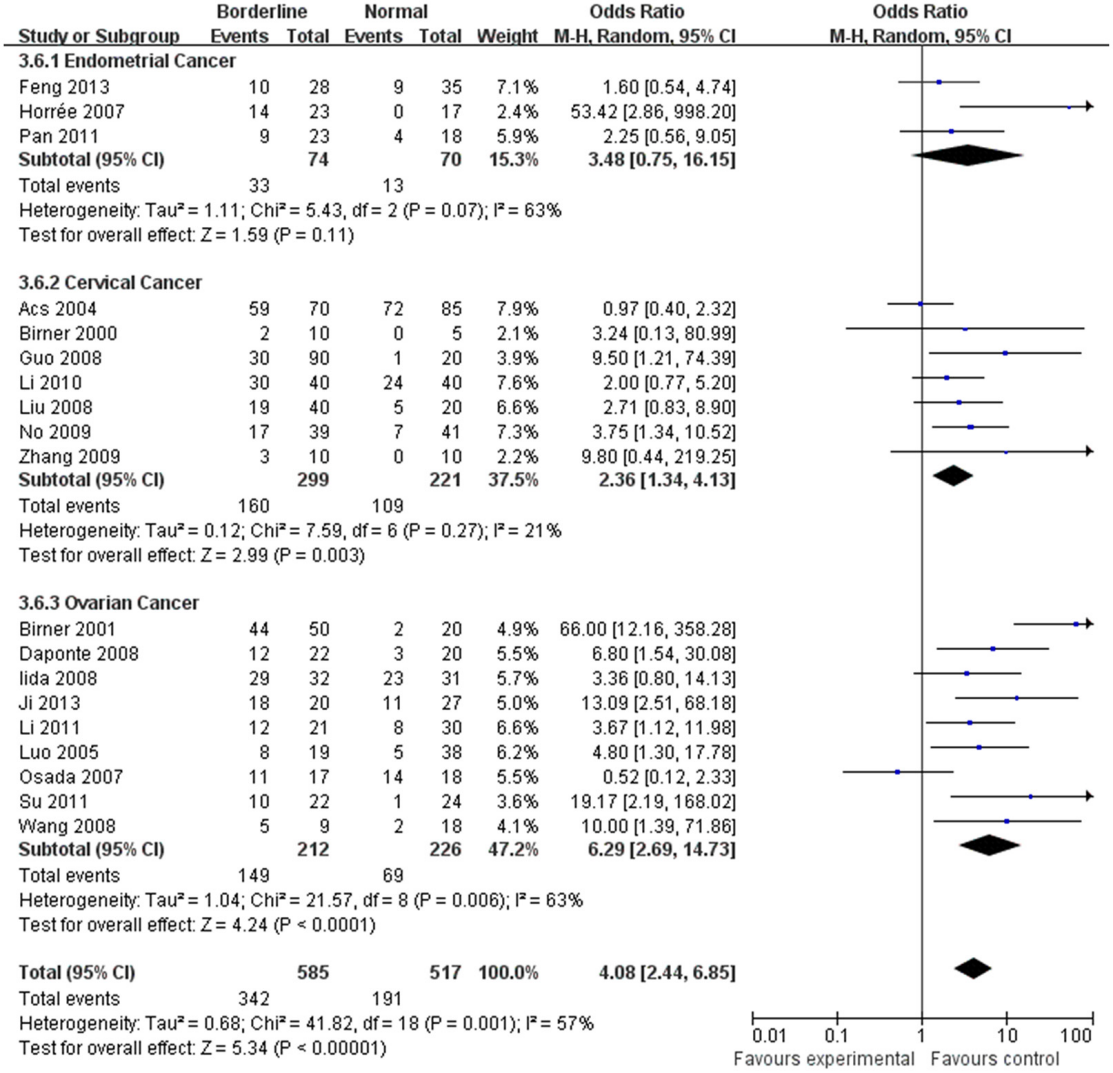
Forest plot of the expression of HIF-1α in borderline versus that in nomal tissue. (*I* ^2^ = 57%).

**Fig 5:**
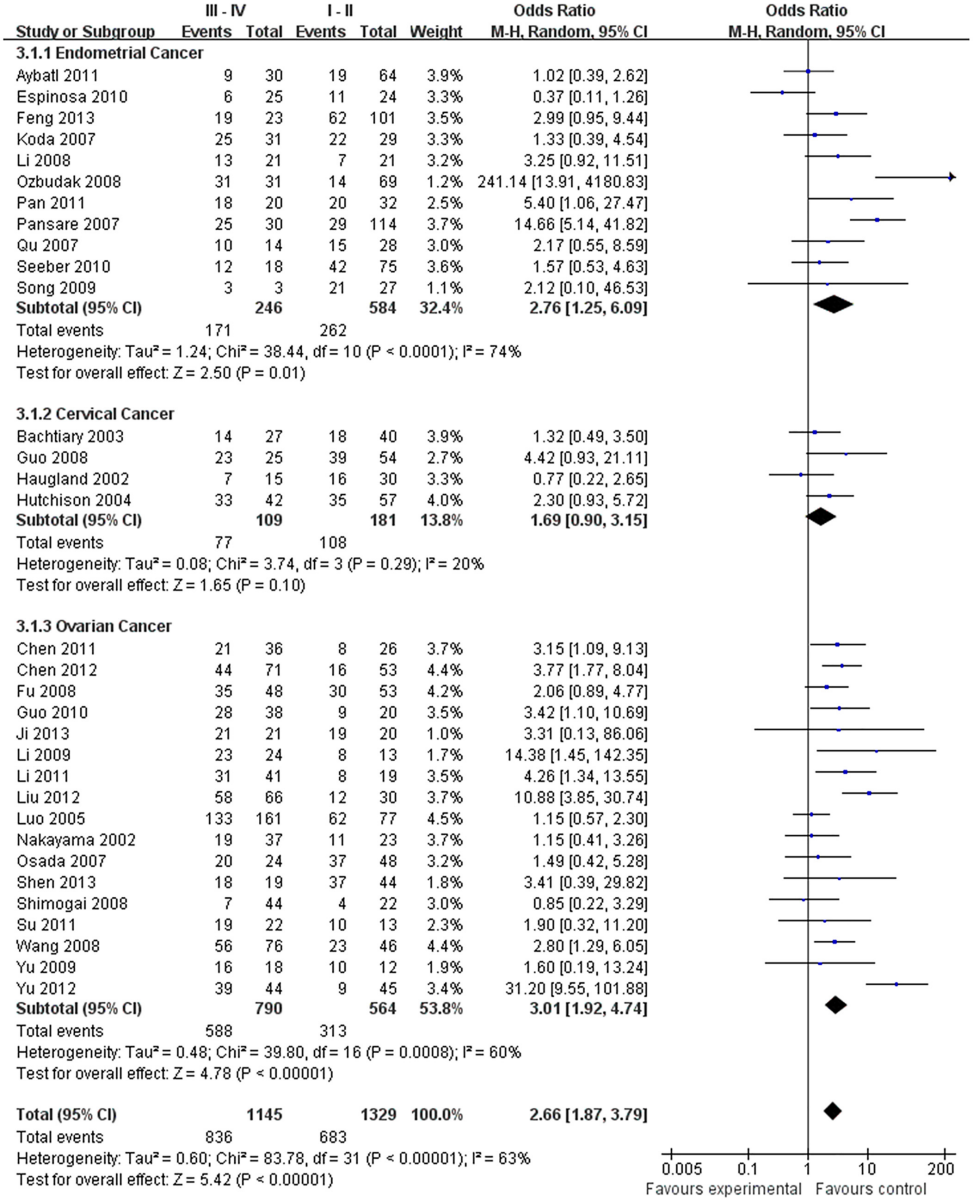
Forest plot of association between HIF-1α expression and FIGO stage. (*I* ^2^ = 63%).

**Fig 6:**
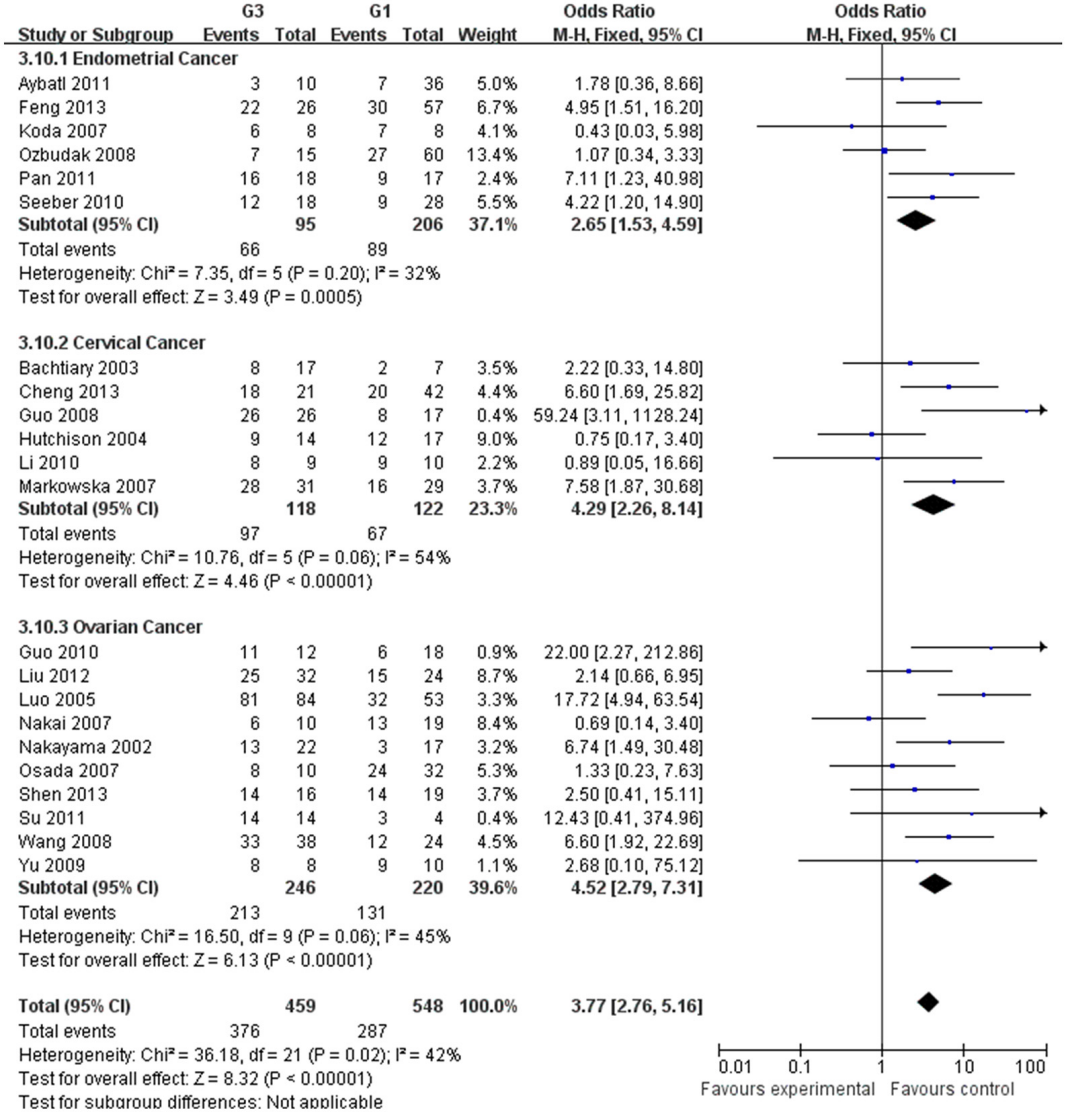
Forest plot of the expression of HIF-1α in Grade 3 tissue versus that in Grade 1 tissue. (*I* ^2^ = 42%).

**Fig 7:**
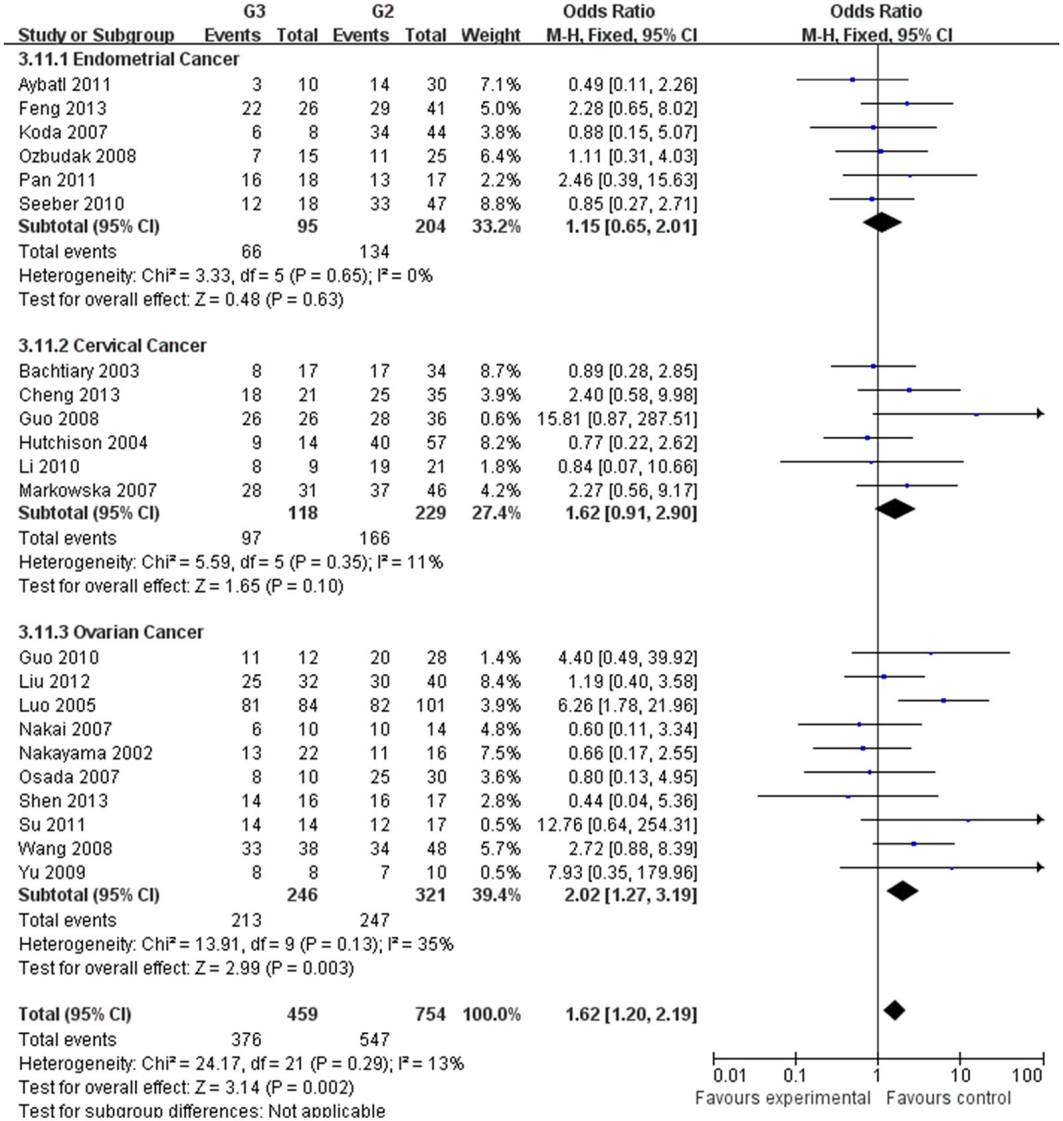
Forest plot of the expression of HIF-1α in Grade 3 tissue versus that in Grade 2 tissue. (*I* ^2^ = 13%).

**Fig 8:**
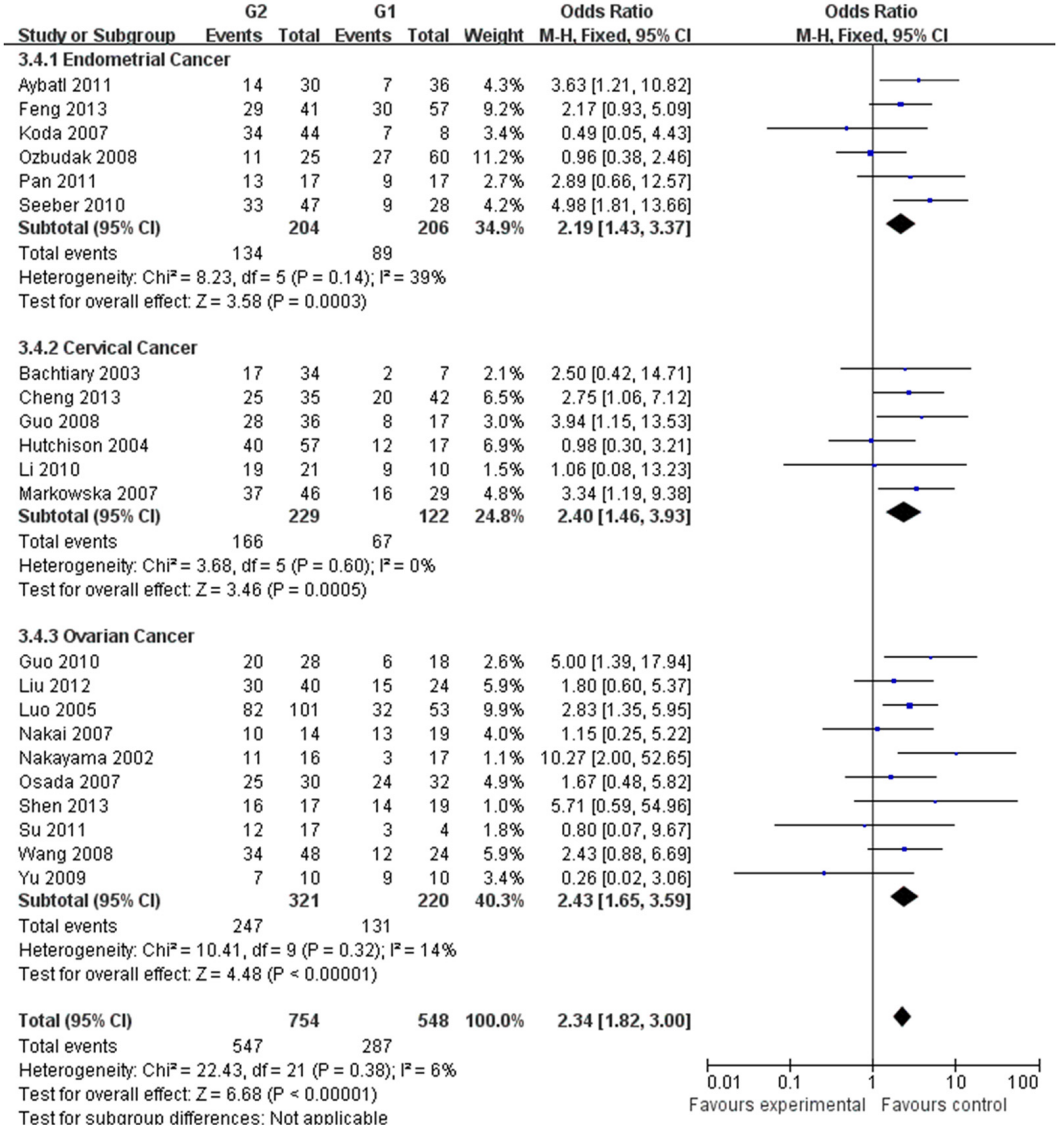
Forest plot of the expression of HIF-1α in Grade 2 tissue versus that in Grade 1 tissue. (*I* ^2^ = 6%).

**Fig 9:**
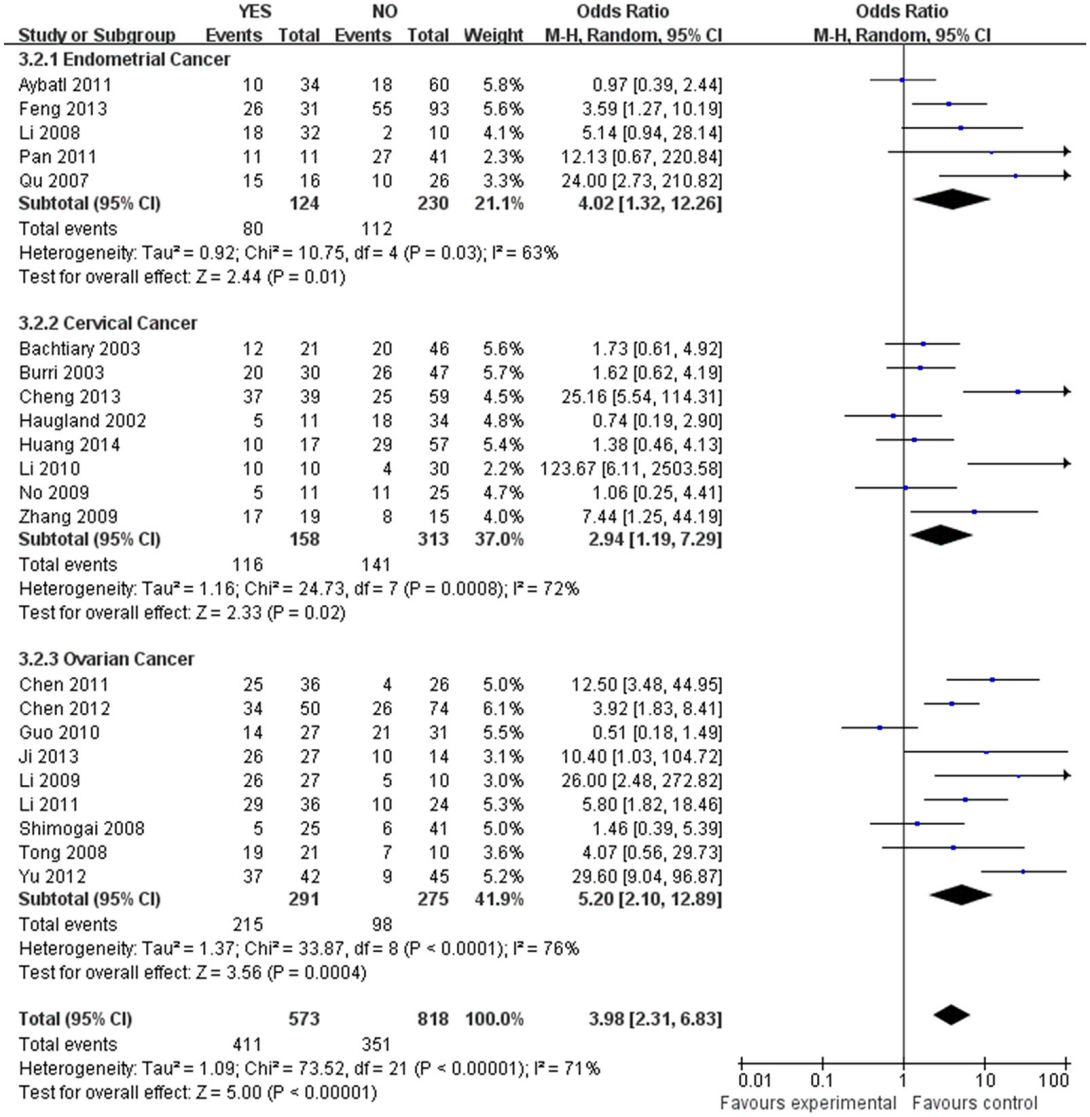
Forest plot of association between HIF-1α expression and lymph node metastasis. (*I* ^*2*^ = 71%).

The estimated pooled OR for all studies showed a significantly increased risk of malignant progression (cancer vs. borderline: OR, 2.70; 95% CI, 1.69–4.31, cancer vs. normal: OR, 9.59; 95% CI, 5.97–15.39, borderline vs. normal: OR, 4.13; 95% CI, 2.43–7.02, Figs 2–4, all *p*<0.05), higher FIGO stage (III–IV vs. I–II: OR, 2.66; 95% CI, 1.87–3.79, Fig 5, *p*<0.05), higher grade type (Grade 3 vs. Grade 1: OR, 3.77; 95% CI, 2.76–5.16, Grade 3 vs. Grade 2: OR, 1.62; 95% CI, 1.20–2.19, Grade 2 vs. Grade 1: OR, 2.34; 95% CI, 1.82–3.00, Figs 6–8, all *p*<0.05) and lymph node metastasis (yes vs. no: OR, 3.98; 95% CI, 2.10–12.89, Fig 9, *p*<0.05) in patients with positive HIF-1α expression. To explore potential sources of heterogeneity, we conducted subgroup analyses considering tumor types of gynecological cancer including endometrial, cervical and ovarian cancer. Almost all subgroup analyses maintained the positive association except the analysis of endometrial (borderline vs. normal: OR, 3.48; 95% CI, 0.75–16.15, Fig 4, *p* = 0.11, Grade 3 vs. Grade 2: OR, 1.15; 95% CI, 0.65–2.01, Fig 7, *p* = 0.63.) and cervical cancer (Grade 3 vs. Grade 2: OR, 1.62; 95% CI, 0.91–2.90, Fig 3, *p* = 0.10).

### HIF-1α expression and 5-year DFS rate, 5-year OS rate

**Fig 10:**
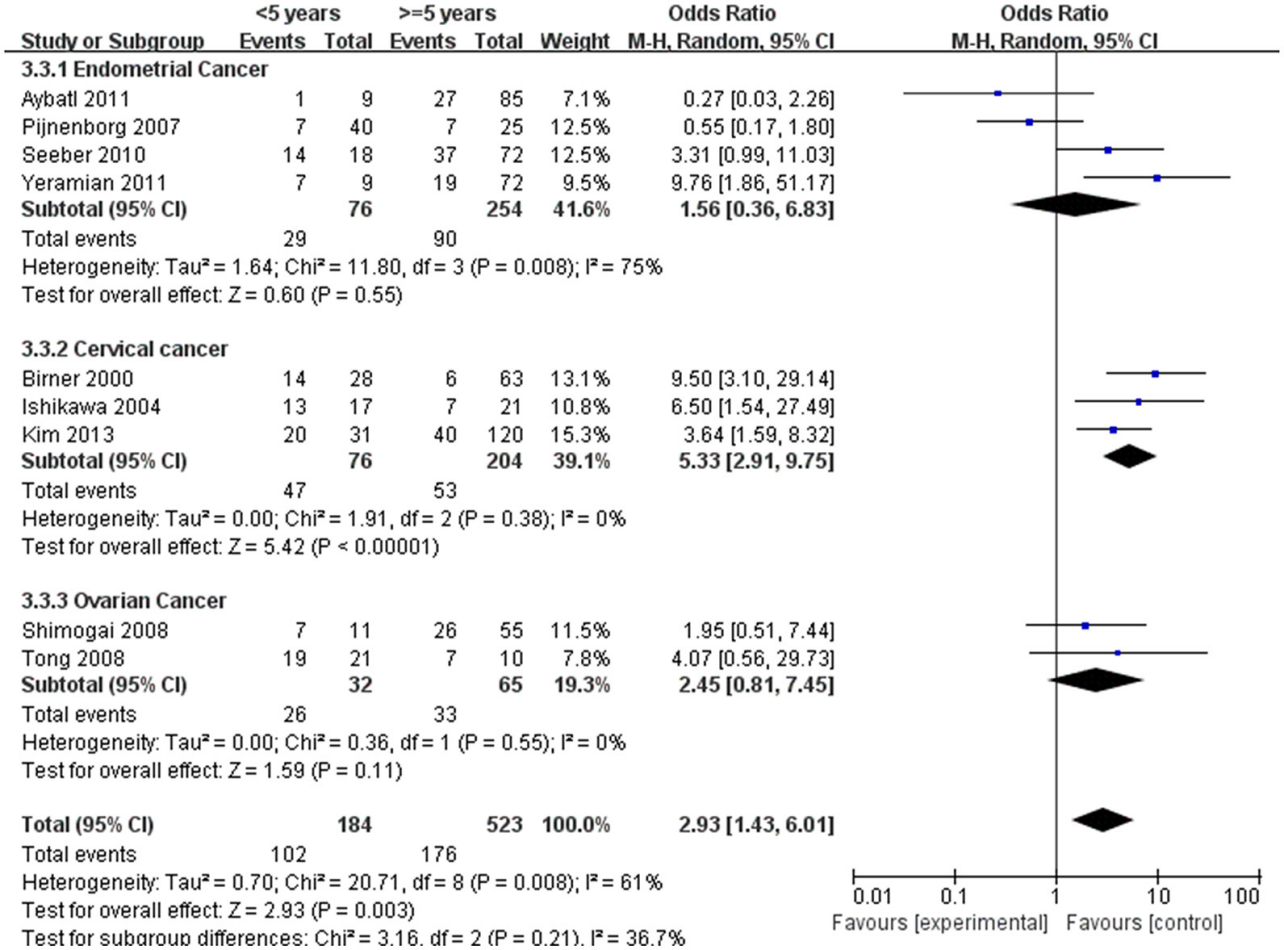
Forest plot of association between HIF-1α expression and 5-years desease free survival rate. (*I* ^*2*^ = 61%).

**Fig 11:**
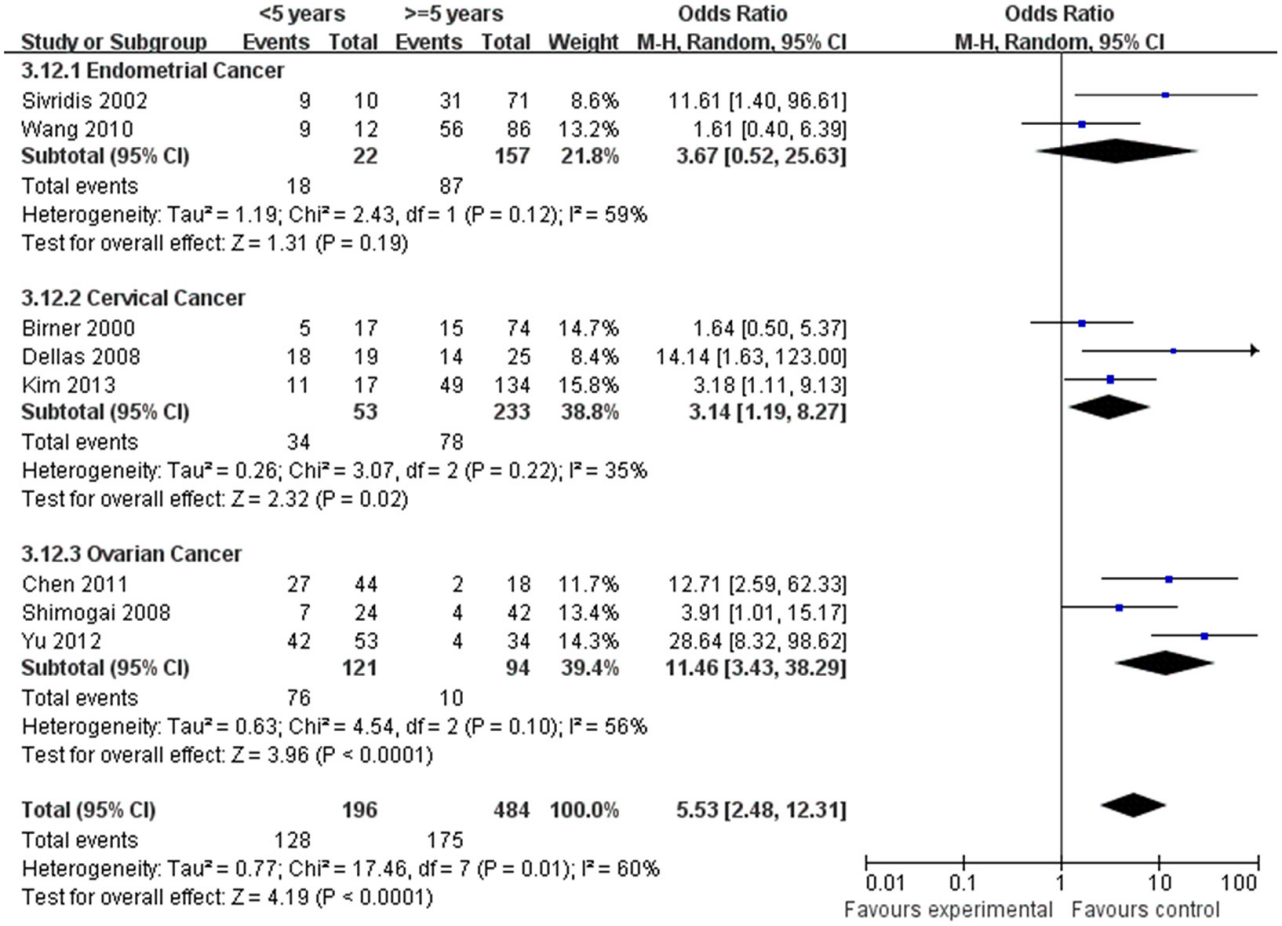
Forest plot of association between HIF-1α expression and 5-years overall survival rate. (*I* ^*2*^ = 60%).

The estimated pooled OR for 14 studies on the prognostic value of HIF-1α expression showed the positive expression of HIF-1α were associated with lower 5-year DFS and OS rate (<5 years vs. ≥5 years, Figs 10 and 11, *p*<0.05), the OR (95% CI) was 2.93(1.43,6.01), 5.53(2.48,12.31), respectively. To explore potential sources of heterogeneity, we conducted subgroup analyses. However, the subgroup of endometrial (DFS: OR, 1.56; 95% CI, 0.36–6.83, Fig 10, *p* = 0.55, OS: OR, 3.67; 95% CI, 0.52–25.63, Fig 11, *p* = 0.19) and ovarian cancer (DFS: OR, 2.42; 95% CI, 0.80–7.36, Fig 11, *p* = 0.12) did not maintain the positive association.

### Sensitivity analysis

Sensitivity analysis was performed to explore the influence of an individual study on the pooled results by repeating the meta-analysis while omitting some obviously different studies at the time. Statistically similar results were obtained by this procedure, indicating the stability of this meat-analysis (data not shown).

## Discussion

HIF-1α is a key transcription factor that regulates cellular reaction to hypoxia. It is over-expressed in many types of malignancies in response to low oxygen concentration [66], and plays a key role in hypoxic conditions that occur during tumor angiogenesis, invasion and metastasis [67, 68]. In gynecological cancer, HIF-1α has been suggested as an adverse prognostic factor, but conflicting findings do exist [69]. Thus, pooled analysis was performed with available data on the association between HIF-1α expression and clinicopathological variables.

We demonstrated that the expression of HIF-1α in normal tissue was lower than that in borderline or cancer tissue in gynecological cancer, which is in agreement with previous findings from different studies [2, 8, 9, 16, 27, 30, 52, 57, 70]. HIF-1α may be a facilitator of premalignant progression in gynecological cancer. This positive association maintained in most subgroup analyses except in the “borderline vs. normal” of endometrial cancer. This inconsistence may result from a relatively small number of included studies (only three studies were in the subgroup analysis).

Clinicopathologicfeatures including pathological type, tumor stage, and lymph node metastasis are the major facts related to cancer-related prognosis. In our meta-analysis, higher HIF-1α expression was found to be associated with increased risk of lymph node metastasis, higher FIGO stage, higher histological grade, and lower 5-year OS and DFS rate. These findings revealed that HIF-1α could be considered as a hallmark of tumour progression, and a prognostic factor for gynecological cancer. To reveal the mechanisms, several included studies of this meta-analysis reported that HIF-1α is related to many critical aspects of gynecological cancer biology. HIF-1α synthesis could be increased by several growth factors, cytokines and other signaling molecules responsible for stimulating phosphatidylinositol 3-kinase (PI3K) or mitogen-activated protein kinase (MAPK) pathways [38]. The regulated markers of HIF-1α, such as glucose transporter type 1 (GLUT1), carbonic anhydrase 9 (CA9) and c-Met, have been found to be highly associated with poor prognosis in various cancers [38]. HIF-1α also regulates many cancer signaling pathways, including PI3K/AKT/mTOR, Notch, and Myc, to mediate tumor proliferation, invasion and migration [2, 8, 9, 16, 27, 30, 52, 57, 70].

However, the association between HIF-1α and the clinicopathologic features was not observed in subgroup analyses of “Grade 3 vs. Grade 2” in endometrial and cervical cancers. When stratified by cancer type, results of survival analysis were not statistically significant in the “endometrial and ovarian cancer” subgroup. We suggested that besides the heterogeneity of included studies, other factors related to clinicopathologic features of gynecological cancer might contribute to this inconsistence. For example, type I endometrial cancer is often characterized by mutations in tumor suppressor PTEN, while type II endometrial cancer generally contains the mutation of another tumor suppressor p53 [71–74]. In cervical cancer, the overexpression of human papillomavirus (HPV) and the loss of p53 promote tumor invasion and metastasis [75]. Thus, further studies included both HIF-1α and other factors are warranted to validate our findings, and to unravel the mechanism of carcinogenesis and progression in gynecological cancer.

Some limitations should be acknowledged. First, immunohistochemistry was a semiquantitative method, and this may affect the precision of the result. In this meta-analysis, no subgroup survival analysis was performed for different histological subtypes. Differences in primary antibodies, immunohistochemistry staining protocols, evaluation standards, and cut-off values for high HIF-1α expression might contribute to heterogeneity. However, this meta-analysis pooled series of studies and had higher statistical power to make up for this disadvantage to some extent. Further multicenter researches using standardized and quantitative methods are encouraged. Second, this meta-analysis included studies published in between 2001 and 2014. During those 13 years, improved surgical techniques and better perioperative care were developed at more specialized centers. The time-varying therapeutic regimen would be the major source of heterogeneity in cancer-related prognosis. For example, in the survival analysis of the “endometrial and ovarian cancer” subgroup, three studies reported postoperative adjuvant chemotherapy, fourteen studies reported postoperative adjuvant radiotherapy, while others did not provide any information about postoperative adjuvant therapy. Thus, the results of the prognosis analyses should be interpreted with caution. Third, more than half of included studies in this meta-analysis are from Asia. Because of this population bias, our results might not fully reveal the association of HIF-1α and clinicopathological characteristics of patients all over the world. Therefore, patients from a variety of countries should be studied to improve the reliability of our analysis in the near future.

## Conclusions

Despite the limitations of this meta-analysis, we confirmed that HIF-1α is emerging as an important factor in the carcinogenesis of gynecological cancer. HIF-1α is associated with the malignantdegree, FIGO stage, histological grade, lymph node metastasis, 5-years survival rate and recurrence rate of gynecological cancer. We expect that HIF-1α may serve as a reliable tool for early and accurate prediction of cancer and may be a potential therapeutic target for gynecological cancer.

## Supporting Information

S1 PRISMA Checklist PRISMA Checklist. (DOC)

S1 Table Quality assessments of included studies. (DOC)
