# Supplementary material for: Clinicopathological Characteristics of Gynecological Cancer Associated with Hypoxia-Inducible Factor 1α Expression: A Meta-Analysis Including 6,612 Subjects
Source: PLoS One. 2015 May 19;10(5):e0127229. doi: 10.1371/journal.pone.0127229 (PMC4438056; doi:10.1371/journal.pone.0127229)
Supplement: S1 Table — (DOC) [file pone.0127229.s002.doc]

**S1 Table.** Quality assessments of included studies.

| Author | Year (country) | 1 | 2 | 3 | 4 | 5A | 5B | 6 | 7 | 8 | Total |
| --- | --- | --- | --- | --- | --- | --- | --- | --- | --- | --- | --- |
| **Ovarian cancer** |  |  |  |  |  |  |  |  |  |  |  |
| Daponte^14^ | 2008(Greece) | 1 | 1 | 1 | 0 | 0 | 0 | 1 | 1 | 0 | 5 |
| Shimogai^13^ | 2008 (Japan) | 1 | 1 | 1 | 0 | 1 | 0 | 0 | 1 | 0 | 5 |
| Yu^15^ | 2012 (China) | 1 | 1 | 1 | 0 | 1 | 0 | 0 | 1 | 0 | 5 |
| Birner^10^ | 2001 (Austria) | 1 | 1 | 1 | 0 | 1 | 1 | 1 | 1 | 0 | 7 |
| Osada^16^ | 2007 (Japan) | 1 | 1 | 1 | 0 | 0 | 1 | 1 | 1 | 0 | 6 |
| Shen^17^ | 2013 (China) | 1 | 1 | 1 | 0 | 0 | 0 | 0 | 1 | 0 | 4 |
| Su^18^ | 2011 (China) | 1 | 1 | 1 | 0 | 1 | 0 | 1 | 1 | 0 | 6 |
| Y u^19^ | 2009 (China) | 1 | 1 | 1 | 0 | 1 | 0 | 0 | 1 | 0 | 5 |
| Liu^20^ | 2012 (China) | 1 | 1 | 1 | 1 | 1 | 0 | 0 | 1 | 0 | 6 |
| Chen^21^ | 2011 (China) | 1 | 1 | 1 | 1 | 1 | 0 | 1 | 1 | 0 | 7 |
| Fu^22^ | 2008 (China) | 1 | 1 | 1 | 1 | 1 | 0 | 0 | 1 | 0 | 6 |
| Guo^23^ | 2010 (China) | 1 | 1 | 1 | 1 | 0 | 0 | 0 | 1 | 0 | 5 |
| Naka^26^ | 2007 (Japan) | 1 | 1 | 1 | 0 | 1 | 1 | 0 | 1 | 0 | 6 |
| Ji^25^ | 2013 (China) | 1 | 1 | 1 | 1 | 1 | 0 | 0 | 1 | 0 | 6 |
| Nakayama^26^ | 2002 (Japan) | 1 | 1 | 1 | 0 | 1 | 1 | 1 | 1 | 0 | 7 |
| Iida^27^ | 2008 (Japan) | 1 | 1 | 1 | 0 | 1 | 0 | 0 | 1 | 0 | 5 |
| Chen^28^ | 2012 (China) | 1 | 1 | 1 | 1 | 1 | 0 | 1 | 1 | 0 | 7 |
| Li^29^ | 2011(China) | 1 | 1 | 1 | 1 | 1 | 0 | 0 | 1 | 0 | 6 |
| Wong^30^ | 2003(USA) | 1 | 1 | 1 | 0 | 1 | 0 | 0 | 1 | 0 | 5 |
| Luo^31^ | 2005(China) | 1 | 1 | 1 | 1 | 1 | 0 | 0 | 1 | 0 | 6 |
| Wang^32^ | 2008(China) | 1 | 1 | 1 | 1 | 1 | 0 | 0 | 1 | 0 | 6 |
| Tong^34^ | 2008(China) | 1 | 1 | 1 | 0 | 1 | 1 | 0 | 1 | 0 | 6 |
| Li^33^ | 2009(China) | 1 | 1 | 1 | 1 | 1 | 0 | 0 | 1 | 0 | 6 |
| Miyazawa^35^ | 2009(Japan) | 1 | 1 | 1 | 0 | 1 | 0 | 0 | 1 | 0 | 5 |
| Yasuda^36^ | 2008(Japan) | 1 | 1 | 1 | 0 | 1 | 0 | 0 | 1 | 0 | 5 |
| **Cervical cancer** |  |  |  |  |  |  |  |  |  |  |  |
| Cheng^37^ | 2013(China) | 1 | 1 | 1 | 1 | 1 | 0 | 1 | 1 | 0 | 7 |
| Kim^38^ | 2013(Korea) | 1 | 1 | 1 | 1 | 0 | 0 | 1 | 1 | 0 | 6 |
| Huang^39^ | 2014(China) | 1 | 1 | 1 | 1 | 1 | 0 | 1 | 1 | 0 | 7 |
| Dellas^40^ | 2008(Germany) | 1 | 1 | 1 | 1 | 0 | 0 | 1 | 1 | 0 | 6 |
| Birner^8^ | 2000(Austria) | 1 | 1 | 1 | 0 | 0 | 0 | 1 | 1 | 0 | 5 |
| Bachtiary^12^ | 2003(Austria) | 1 | 1 | 1 | 0 | 0 | 0 | 1 | 1 | 0 | 5 |
| Li^41^ | 2010(China) | 1 | 1 | 1 | 0 | 0 | 0 | 1 | 1 | 0 | 5 |
| Guo^42^ | 2008(China) | 1 | 1 | 1 | 0 | 0 | 0 | 1 | 1 | 0 | 5 |
| Liu^43^ | 2008(China) | 1 | 1 | 1 | 0 | 0 | 0 | 1 | 1 | 0 | 5 |
| Zhang^44^ | 2009(China) | 1 | 1 | 1 | 0 | 0 | 0 | 1 | 1 | 0 | 5 |
| Acs^45^ | 2003(USA) | 1 | 1 | 1 | 1 | 0 | 0 | 1 | 1 | 0 | 6 |
| Hutchison^46^ | 2004(United Kingdom) | 1 | 1 | 1 | 1 | 0 | 0 | 1 | 1 | 0 | 6 |
| No^47^ | 2009(Korea) | 1 | 1 | 1 | 1 | 0 | 0 | 1 | 1 | 0 | 6 |
| Ishikawa^48^ | 2004(Japan) | 1 | 1 | 1 | 1 | 1 | 0 | 1 | 1 | 0 | 7 |
| Haugland^49^ | 2002(Canada) | 1 | 1 | 1 | 0 | 0 | 0 | 1 | 1 | 0 | 5 |
| Burri^50^ | 2003(Switzerland) | 1 | 1 | 1 | 0 | 0 | 0 | 1 | 1 | 0 | 5 |
| Markowska^51^ | 2007(Poland) | 1 | 1 | 1 | 0 | 0 | 0 | 1 | 1 | 0 | 5 |
| **Endometrial**  **cancer** |  |  |  |  |  |  |  |  |  |  |  |
| Ozbudak^52^ | 2008(Turkey) | 1 | 1 | 1 | 0 | 0 | 0 | 1 | 1 | 0 | 5 |
| Feng^53^ | 2013(China) | 1 | 1 | 1 | 0 | 0 | 0 | 1 | 1 | 0 | 5 |
| Espinosa^54^ | 2010(Italy) | 1 | 1 | 1 | 0 | 0 | 0 | 1 | 1 | 0 | 5 |
| Seeber^69^ | 2010(Netherlands) | 1 | 1 | 1 | 0 | 0 | 0 | 1 | 1 | 0 | 5 |
| Pijnenborg^55^ | 2007(Netherlands) | 1 | 1 | 1 | 1 | 1 | 0 | 1 | 1 | 0 | 7 |
| Acs^9^ | 2004(USA) | 1 | 1 | 1 | 1 | 1 | 0 | 1 | 1 | 0 | 7 |
| Pansare^56^ | 2007(USA) | 1 | 1 | 1 | 1 | 0 | 0 | 1 | 1 | 0 | 6 |
| Horrée^57^ | 2007(Netherlands) | 1 | 1 | 1 | 1 | 0 | 0 | 1 | 1 | 0 | 5 |
| Koda^58^ | 2007(Poland) | 1 | 1 | 1 | 0 | 0 | 0 | 1 | 1 | 0 | 5 |
| Aybatli^59^ | 2011(Turkey) | 1 | 1 | 1 | 0 | 1 | 0 | 1 | 1 | 0 | 6 |
| Yeramian^60^ | 2011(Spain and USA) | 1 | 1 | 1 | 1 | 0 | 0 | 1 | 1 | 0 | 6 |
| Li^61^ | 2008(China) | 1 | 1 | 1 | 1 | 0 | 0 | 1 | 1 | 0 | 6 |
| Zhai^62^ | 2007(China) | 1 | 1 | 1 | 0 | 0 | 0 | 1 | 1 | 0 | 5 |
| Pan^63^ | 2011(China) | 1 | 1 | 1 | 1 | 0 | 0 | 1 | 1 | 0 | 6 |
| Song^64^ | 2009(China) | 1 | 1 | 1 | 1 | 0 | 0 | 1 | 1 | 0 | 6 |
| Sivridis^2^ | 2002(Greece) | 1 | 1 | 1 | 1 | 0 | 0 | 1 | 1 | 0 | 6 |
| Wang^65^ | 2010(China) | 1 | 1 | 1 | 1 | 0 | 0 | 1 | 1 | 0 | 6 |

1: with independent validation; 2: consecutive or obviously representative series of cases; 3: community controls; 4: no history of disease; 5A: study controls for age; 5B: study controls for any additional factor (tumor size); 6: structured interview where blind to case/control status; 7: same method of ascertainment for cases and controls; 8: same Non-Response Rate for both groups.
